# Effects of Essential Oils and Fragrant Compounds on Appetite: A Systematic Review

**DOI:** 10.3390/ijms24097962

**Published:** 2023-04-27

**Authors:** Nhi Phuc Khanh Nguyen, Khoa Nguyen Tran, Ly Thi Huong Nguyen, Heung-Mook Shin, In-Jun Yang

**Affiliations:** Department of Physiology, College of Korean Medicine, Dongguk University, Gyeongju 38066, Republic of Korea; npkhanhnhi@dgu.ac.kr (N.P.K.N.); trannguyen053@dgu.ac.kr (K.N.T.); nguyenthihuongly_t58@hus.edu.vn (L.T.H.N.)

**Keywords:** appetite, eating disorder, essential oil, food intake, fragrant compound

## Abstract

Appetite dysregulation is one of the factors contributing to anorexia, bulimia nervosa, obesity, and diabetes. Essential oils or fragrant compounds have been proven to regulate food intake and energy expenditure; hence, this study aimed to summarize their effects on appetite and the underlying mechanisms. The PubMed and Web of Science databases were searched until July 2022. Only two of the 41 studies were performed clinically, and the remaining 39 used animal models. Oral administration was the most common route, and a dosage range of 100–2000 mg/kg for mice or 2–32 mg/kg for rats was applied, with a duration of 12 days to 4 weeks, followed by inhalation (10^−6^–10^−3^ mg/cage or 10^−9^–10^−2^ mg/cm^3^ within 1 h). Approximately 11 essential oil samples and 22 fragrant compounds were found to increase appetite, while 12 essential oils and seven compounds decreased appetite. These fragrant components can exert appetite-regulating effects via leptin resistance, the activity of sympathetic/parasympathetic nerves, or the mRNA expression of neuropeptide Y (NPY)/agouti-related protein (AgRP), cocaine- and amphetamine-regulated transcript (CART)/proopiomelanocortin (POMC) in the hypothalamus. Fragrance memory and cognitive processes may also play roles in appetite regulation. The findings of this study accentuate the potential of essential oils and fragrant compounds to regulate appetite and eating disorders.

## 1. Introduction

From a biological perspective, appetite refers to the internal driving force or inclination to seek, select, and consume food, which is often influenced by physiological, psychological, and environmental factors [1,2,3,4]. Appetite is mainly tuned by homeostatic and hedonic mechanisms [5]. Homeostatic control refers to regulating food intake to maintain energy needs. For example, when the body is metabolically hungry, ghrelin is produced, and it signals the arcuate nucleus (ARC) of the hypothalamus for neuropeptide Y (NPY) or agouti-related peptide (AgRP) release, which stimulates hunger and food intake. Leptin signals fat-filled adipocytes and stimulates the proopiomelanocortin (POMC) or cocaine- and amphetamine-regulated transcript (CART) neurons to reduce food intake [6]. In addition, the homeostatic appetite mechanism also involves gut hormones such as glucagon-like peptide-1 (GLP-1) and cholecystokinin (CCK) that regulate appetite and food intake by reducing hunger and increasing satiety [7]. Contrarily, the hedonic mechanism refers to reward, emotional, and cognitive factors and is driven by visual cues and the smell, or taste of palatable food rather than metabolic signals [5]. The nucleus accumbens, ventral pallidum, and brainstem are the major areas that generate hedonic impact in the food reward system. In contrast, the hippocampus (HPC), prefrontal cortex, and amygdala function together to govern memory and attention for the neural control of appetite [8,9].

Homeostatic and hedonic mechanisms are distinct networks that interact with one another to regulate appetite [10]. Once appetite is dysregulated, it may result in eating disorders such as anorexia nervosa and bulimia nervosa, leading to obesity, diabetes mellitus, osteoporosis, cardiovascular disturbances, or gastrointestinal disorders [11,12,13,14,15]. Although patients with abnormal appetites and subsequent eating disorders have been treated with several types of medications, adverse side effects have limited their widespread use. Lorcaserin and phentermine are drugs used by patients with obesity to manage their increased appetite. Lorcaserin promotes the expression of 5-hydroxytryptamine 2C receptors on the ARC POMC of the hypothalamus, whereas phentermine triggers norepinephrine release and sympathetic nerve activity (SNA), thereby reducing appetite [16,17]. However, these drugs can lead to headaches, nervousness, gastrointestinal disturbances, high blood pressure, blurred vision, valvular heart disease, and insomnia [16,18]. Megestrol acetate is prescribed for older people and chemotherapy patients who face appetite loss. While the precise action of this drug has not been elucidated, it is suggested to target ARC NPY or calcium channels in the satiety center of the ventromedial hypothalamus [19]. However, megestrol acetate’s effects could also result in hyperglycemia, hypoglycemia, venous thromboembolism, and hepatic dysfunction [20,21]. Fluoxetine and lisdexamfetamine are the only two drugs approved by the US Food and Drug Administration for the normalization of bulimia nervosa and binge eating disorder by inhibiting the presynaptic reuptake of serotonin, norepinephrine, and dopamine, respectively. However, the side effects of these interventions include insomnia, tremor, dry mouth, and headaches [22,23]. These situations highlight the urgency of developing more alternative medicines for treating impaired appetite control and related diseases.

One of the potential materials for appetite regulation is essential oils. Essential oils are a mixture of secondary metabolites from aromatic and medicinal plants usually obtained by steam distillation or hydro distillation [24]. Essential oils are colorless, volatile liquids, soluble in organic solvents, and most have strong odors with complex natural mixtures of 20–60 components [25]. In a pure essential oil, the volatile fraction accounts for approximately 90–95% of the total weight, with the major compounds being benzenoids, phenylpropanoids, and terpenoids; the other 5–10% are nonvolatile residues containing fatty acids, flavonoids, carotenoids, and hydrocarbons [26]. The diversity in chemical compositions gives essential oils a wide range of biological activities. For example, essential oils from *Clinopodium nepeta*, *Origanum vulgare*, and *Foeniculum vulgare* had strong antibacterial activity, while *Melissa officinalis* L., *Mentha piperitae* L., and *Ocimumbasilicum* L. can be applied as antioxidants [27]. Regarding anticancer effects, essential oils of *Abies koreana* E.H. Wilson and *Abies alba* Mill. and their fragrant compounds, including α-pinene, β-pinene, β-myrcene, limonene, and camphene, inhibited the growth of breast cancer cell lines. The antitumor effects in mice of essential oil constituents such as hinokitiol, geraniol, and citronellol have also been documented [28]. Notably, increasing studies have reported the application of essential oils and their compounds in treating mood disorders such as depression, anxiety, and sleep disorders. Major components of essential oils are volatile lipophilic compounds, which favor their straightforward absorption via the blood-brain barrier (BBB) or stimulation of the olfactory system. Signals conveyed to the limbic and hypothalamic regions by olfactory sensory neurons can be projected to higher circuits such as the prefrontal cortex, amygdala, hypothalamus, basal ganglia, and HPC [29,30]. These regions play critical roles in mood and emotional processing. *Chamaemelum nobile* L. essential oil or its main component, α-pinene, was shown to reduce depression-like behaviors in rats by increasing the expression of parvalbumin mRNA in the HPC [31]. The anxiolytic effects of limonene in citrus essential oil, caryophenol in rose essential oil, and linalool in lavender essential oil have also been reviewed [24]. Compound Anshen essential oil, a blended formula of lavender, sweet orange, sandalwood, frankincense, orange blossom, rose, and agarwood essential oils, can increase 5-hydroxytryptamine and gamma-aminobutyric acid levels in the brain to exert its sleep-promoting effect [32]. Due to their ability to influence multiple brain regions and emotional control, the potential application of essential oils in appetite regulation, especially via hedonic eating, is extremely promising.

Despite multiple reviews on other pharmacological properties of essential oils, attempts to comprehensively review their appetite-regulating effects are lacking. To our knowledge, the research trend on the effects of essential oils on appetite started in the 2000s; since the 2010s, new molecular mechanisms and relevant brain regions have gradually been revealed but are not fully understood [33,34]. In addition, various inconsistent results have been reported because of the diversity of essential oils, along with differences in dosage, compositions, animal models, and routes of administration. Therefore, we hereby conduct this systematic review, aiming to summarize the effects of different essential and fragrant compounds on appetite and food intake, present differences in dosages, models, and routes of delivery among original studies, and discuss defined and potential mechanisms underlying the actions of essential oils and fragrant compounds on appetite. This review is expected to suggest potential essential oils, fragrant compounds, and important mechanisms for future research addressing appetite dysregulation and related eating disorders.

## 2. Methods

### 2.1. Search Strategies

A systematic review was constructed in accordance with the updated PRISMA 2020 guidelines [35]. The authors searched papers on PubMed and Web of Science published until 10 July 2022, using the following search terms:

- For PubMed: ((appetite[MeSH Terms]) OR (food intake[MeSH Terms]) OR (food consumption[MeSH Terms])) AND ((aroma[MeSH Terms]) OR (aromas[MeSH Terms]) OR (scent[MeSH Terms]) OR (scents[MeSH Terms]) OR (fragrance[MeSH Terms]) OR (fragrances[MeSH Terms]) OR (smell[MeSH Terms]) OR (aromatherapy[MeSH Terms]) OR (aromatherapies[MeSH Terms]) OR (volatile oils[MeSH Terms]) OR (essential oils[MeSH Terms]) OR (essential oil[MeSH Terms])).
- For Web of Science (All Fields): (appetite OR food intake OR food consumption) AND (aroma OR aromas OR scent OR scents OR fragrance OR fragrances OR smell OR aromatherapy OR aromatherapies OR volatile oils OR essential oils OR essential oil).

Two authors independently assessed the title, abstract, and full text of the studies with the following exclusion criteria: (1) review paper; (2) conference abstract; (3) not appetite study; (4) not fragrance study; (5) neither appetite nor fragrance study; and (6) full texts not accessible. All qualified studies examined the effects of essential oils or fragrant compounds on food intake or appetite. Any disagreements between the two researchers were resolved via consensus with a third independent researcher.

### 2.2. Data Extraction and Quality Assessment

We thoroughly evaluated the selected studies and extracted the following data: name of essential oil and its main components/fragrant compounds; route, dosage, and duration of administration; the model being employed; and effect and underlying mechanism of the essential oil or fragrant compound.

Once information extraction was completed, the methodological quality of individual studies was assessed by two independent researchers using a 10-item checklist refined from previously published criteria [36]. One point was granted for studies satisfying each of the following criteria: (1) description of the sampling procedure or compound manufacturer; (2) mention of essential oil compositions; (3) detailed description of interventions; (4) use of positive controls; (5) specific mention of strains/species of animals used; (6) mention of test subject age; (7) mention of test subject weight; (8) mention or explanation of underlying mechanisms; (9) statement of potential conflict of interests; and (10) peer-reviewed publication.

## 3. Results

**Figure 1:**
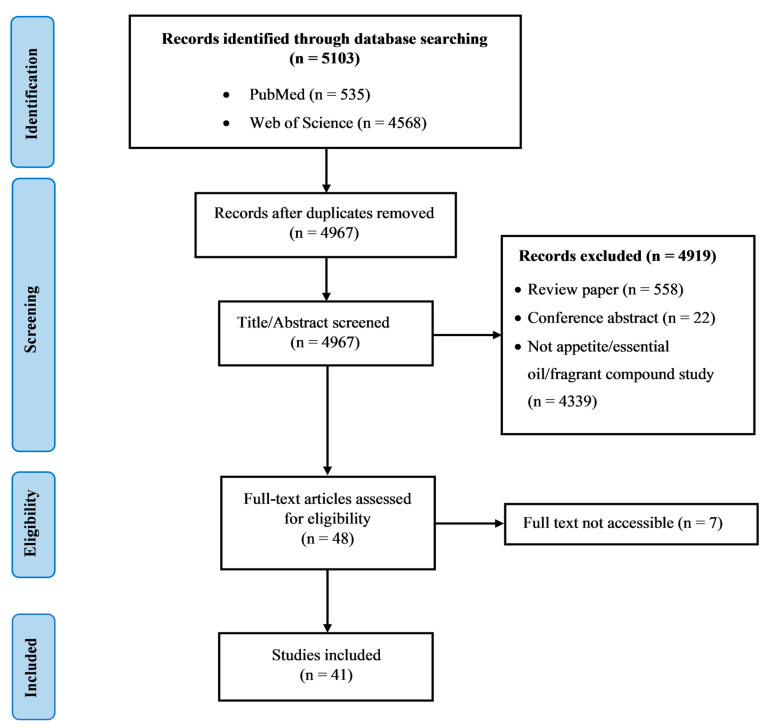
Flow chart of study selection.

Out of 5103 potential records found in both databases, 136 duplicates were removed. The title, abstract, and full-text assessment process eliminated 4926 records that did not meet the eligibility criteria. Ultimately, 41 articles were included in this study. Figure 1 illustrates the flowchart of study selection for this review.

### 3.1. Quality Assessment

The quality assessments of the selected studies are presented in Table 1. The quality scores of the included articles range from 4 to 10, with an average of 7. Among the 41 studies, out of a total score of 10 points, one study scored 10, and five studies scored 9, while the lowest points were recorded in three studies. Fifteen studies scored 7, which was the most predominant score. In terms of assessment criteria, most papers mention the description of samples (origin, sampling method) and model details (species/strains, ages). All of the included studies describe the administration routes and are peer-reviewed. Of the 41 papers, only 14 use positive controls, and 10 explain the underlying mechanisms.

### 3.2. Essential Oil and Fragrant Compound Preparation

Steam distillation for capturing volatile components is the most common strategy for obtaining essential oils. To prepare curry, nutmeg, clove, cinnamon, and fennel essential oils, dried powder or small slices of herbs were mixed with water and hydro-distilled for 3–4 h using the Clevenger apparatus, and the oils were captured in n-hexane. Essential oils were obtained by drying over anhydrous sodium sulfate, followed by n-hexane evaporation. For extracting lavender essential oil (*Lavandula angustifolia*), *Satureja khuzistanica*, and *Hyptis martiusii* Benth., n-hexane was not used as a solvent for dissolving the desired essential oils [47,50,64]. Additionally, in the case of *Aquilaria crassna*, *Amomum villosum* Lour., Cang-ai, or Arq Zeera essential oils (a distillate prepared from *Trachyspermum ammi* L., *Zingiber officinale* Roxb., *Carum carvi* L., and *Cuminum cyminum* L.), the dried materials were macerated with distilled water for a period lasting between 1 h and one week before steam distillation [51,58,60,74]. In some cases, essential oils and fragrant compounds were purchased rather than extracted. The materials were stored at 4 °C or −20 °C for further experiments.

### 3.3. Administration Methods

Several administration routes, including inhalation, injection, intranasal administration, aquarium mixing, and oral delivery (including oral gavage and mixing with food/water methods), were used in the 41 selected studies for delivering essential oils and fragrant compounds. Clinically, one study employed inhalation at a dose of 0.1 mL, while the intranasal method was used in another study at a dose of 100 µL for 1 min. To investigate the effects on appetite in vivo, the common methods used were oral administration (23 studies) of essential oils and fragrant compounds in animal models, followed by inhalation (14 studies), injection (3 studies), and mixing in aquariums (1 study). The frequently employed oral dose range was 100–2000 mg/kg for mice and 2–32 mg/kg for rats, with a duration of 12 days to 4 weeks. In terms of inhalation, mice usually received treatment at 10^−6^–10^−3^ mg/cage or 10^−9^–10^−2^ mg/cm^3^ within 1 h, whereas, in most cases, rats were treated with samples at 100–100,000 times suspended in water within 6–12 weeks.

### 3.4. Effects of Essential Oils and Fragrant Compounds on Appetite

Among the 41 studies, 11 examined the effects of essential oils and related fragrant components, while 21 and 9 studies only focused on essential oils and fragrant compounds, respectively. Terpenes and aromatic compounds, two biosynthetic families, are frequently found as ingredients in these essential oils. Among the 40 essential oils and 46 fragrant compounds, 30 samples promoted appetite, 18 samples exhibited appetite-suppressing effects, and 33 did not show any impact on appetite. In addition, five materials exerted different appetite-related effects depending on the experimental conditions.

#### 3.4.1. Effects of Essential Oils on Appetite in Clinical Studies

Two clinical studies have investigated the appetite-regulating effects of essential oils in humans. Olfactory stimulation with black pepper essential oil, a strong appetite stimulant, for 1 min before every meal increased oral intake and improved swallowing movement in pediatric patients receiving long-term enteral nutrition for neurological disorders [41]. In another clinical study of college-going women, participants were introduced to 12 digitally colored photographs of chocolate foods, and during the retention period after each image, they rated their craving levels for chocolate while inhaling 1/10 mL of essential oils. The results showed that the inhalation of vanilla essential oil, which is considered a sweet scent, increased the rating score for chocolate craving levels compared with the control condition, while the inhalation of Slique Essence, a combination of citrus and mint scents, exhibited the opposite effect [52] (Table 2).

#### 3.4.2. Essential Oils and Fragrant Compounds with Appetite-Enhancing Effects

Animal studies have also demonstrated that treatment with essential oils and fragrant compounds can improve appetite and food intake. Inhalation of curry essential oil or its components (trans-cinnamaldehyde, eugenol, and trans-anethole) at a dose of 4.5 × 10^−4^ mg/cage significantly increased food intake in ddY mice [53]. Similarly, nutmeg essential oil (7.4 × 10^−7^ mg/cm^3^) and its derived compounds, myristicin and methyl eugenol (7.4 × 10^−9^ mg/cm^3^), also exerted appetite-enhancing effects in ddY mice [65]. Another study indicated that cinnamon, clove, and fennel essential oils increased food intake in mice. The fragrant compounds from the cinnamon essential oil (trans-cinnamaldehyde, 3-phenylpropionaldehyde, cinnamyl acetate, 2-methoxycinnamaldehyde, and trans-cinnamyl alcohol), clove essential oil (eugenol, eugenol acetate, methyl salicylate, and benzyl benzoate), and fennel essential oil (trans-anethole and p-anisketone) also showed similar effects on food consumption [67]. Olfactory stimulation (15 min daily for 33 days) with lavender essential oil (suspended in 100,000 volumes of water) or its main component linalool (suspended in 5000 volumes of water) significantly increased food intake and body weight in rats [37]. A study suggested that inhalation of trans-cinnamaldehyde, 1-phenyl-2-butanone, and benzylacetone significantly increased food intake in ddY mice [54]. In a similar study, inhalation of eugenol, vanillin, and ethyl vanillin also exerted appetite-enhancing effects in mice [61]. Inhaling 2,5-dimethyl-4-hydroxy-3(2H)-furanone (DMHF), a fragrant compound generated by the Maillard reaction, for two to six weeks, significantly increased the dietary intake in Wistar rats [69] (Table 3 and Table 4).

Certain essential oils also exhibited appetite-enhancing effects in pathological animal models, including chronic unpredictable mild stress, 5-fluorouracil-induced intestinal mucositis, and high-fat diet (HFD) rat models. The findings showed that long-term inhalation of Cang-ai essential oil significantly improved stress-induced reductions in food intake and body weight in Sprague Dawley rats [74]. Similar results were observed in 5-fluorouracil-induced intestinal mucositis and HFD rat models, in which *Amomum villosum* Lour. and *Zingiber zerumbet* essential oils and their main active constituents normalized the decrease in food consumption [46,58]. Studies on the appetite-related effects of essential oils and fragrant compounds have also been conducted on livestock. Piglets fed a diet containing fennel seed (*Foeniculi aetheroleum*) essential oil (100 mg/kg) for three weeks showed an increase in food intake [39]. Young bulls showed significantly higher food intakes of dry matter and other ingredients when fed clove or cinnamon essential oils in a dose-dependent manner [56] (Table 3 and Table 4).

#### 3.4.3. Essential Oils and Fragrant Compounds with Appetite-Reducing Effects

Fourteen animal studies have suggested the suppressive effects of essential oils and fragrant compounds on appetite and food consumption. Oral administration of the essential oil of *Croton zehntneri* or its component trans-anethole (250 mg/kg) for 10 weeks resulted in a slight but significant decrease in food consumption without any significant changes in body weight in Wistar rats [63]. In a similar study, oral treatment with the essential oil of *Hyptis martiusii* (100 and 500 mg/kg) for 30 days did not affect body weight gain but lowered food intake in both male and female Swiss mice [47]. In contrast, exposure to microencapsulated citral (15,600 ppm) in the feed decreased food consumption and reduced body weight in F344/N rats [33]. Inhalation of grapefruit (*Citrus paradisii*) essential oil and its major compound limonene (15 min, thrice per week for six weeks) remarkably reduced food consumption and body weight in Wistar rats [38]. Similarly, olfactory stimulation with *Osmanthus fragrans* essential oils for 13 days increased the latency to start eating and decreased food intake and body weight in rats [34] (Table 5 and Table 6).

The downregulated appetite effects of essential oils and their compounds were also investigated in HFD models. In Sprague-Dawley rats fed an HFD, inhalation of 1% β-citronellol for five weeks significantly reduced food consumption [48]. Similarly, 30-min inhalation of 0.3% patchouli (*Pogostemon cablin* Benth.) essential oil for 12 weeks reduced food intake and body weight in HFD-fed rats [66]. Oral administration of Arq Zeera and its major compound, thymol, for four weeks significantly reversed HFD-induced increases in food intake and body weight gain in Wistar rats [60]. Lime (*Citrus aurantifolia*) essential oils suppressed food consumption and reduced body weight in both ketotifen-induced weight gain and normal mice [44] (Table 5 and Table 6).

Supplementation with pine essential oil and 1,8-cineole (a common aromatic compound) in the diet significantly decreased food consumption in western grey kangaroos [72]. Piglets fed diets containing caraway seed (*Carvi aetheroleum*) or fennel seed (*Foeniculi aetheroleum)* essential oils (100 mg/kg) showed a decrease in food intake [39]. In pigs, supplementing the basal diet with an encapsulated blend of essential oils from citrus fruit extract, cinnamon, oregano, thyme, and capsicum reduced food intake without changing body weight [57]. Dietary supplementation with thyme essential oil (400 ppm) decreased food intake, with no significant change in body weight gain in Japanese quails [59]. Flies simultaneously exposed to D-limonene and sucrose for 10 min showed significantly decreased appetite compared with flies exposed to sucrose alone [42] (Table 5 and Table 6).

#### 3.4.4. Essential Oils and Fragrant Compounds with No Effect on Appetite

Twenty-one animal studies have suggested that certain essential oils and fragrant compounds do not affect appetite or food consumption. In Sprague-Dawley rats fed an HFD, inhalation of 1% citronella essential oil or its related compound R-citronellal for five weeks did not affect food consumption or weight gain [48]. Inhaled ginger or geranium essential oil did not affect food intake in ddY mice [53,54]. Similarly, inhalation of fragrant compounds, including β-caryophyllene, butylbenzene, 6-methyl-5-hepten-2-one, benzaldehyde, (R)-linalool, elemicin, isoeugenol, coumarin, estragole, p-anisaldehyde, and safrole, did not cause any changes in food consumption in mouse models [53,54,61,65,67]. Intraperitoneal injection of fragrant compounds, such as vanillin, eugenol, or benzylacetone (0.01–1 μg/kg), did not affect food intake in mice [54,61]. Oral administration of *Lavandula angustifolia* (2000 mg/kg) or *Aquilaria crassna* essential oil (100 and 500 mg/kg) for 21 and 28 days, respectively, did not induce any significant changes in food consumption or body weight in either male or female Swiss mice [51,64]. Oral administration of cuminaldehyde (6 mg/kg) for four weeks did not affect HFD-induced increases in food intake and body weight gain in Wistar rats [60]. Sprague-Dawley rats fed diets containing ionone epoxide, a fragrant material (20, 40, and 80 mg/kg), for 90 days did not show any significant changes in dietary intake or body weight gain [71]. Chronic exposure to diets mixed with essential oil from *Minthostachys verticillata* did not alter dietary consumption or body weight in either male or female Wistar rats [49] (Table 7 and Table 8).

Broiler chicken-fed diets containing peppermint essential oil (200 and 400 mg/kg) showed no change in food intake or body weight [45]. The addition of *Satureja khuzistanica* essential oil (200 and 500 mg/L) to drinking water did not alter food intake but decreased weight gain in Arian broiler chicks aged 29 to 35 days [50]. In a similar study, diets supplemented with lavender and/or mint essential oils (250 mg/kg) did not affect body weight or food intake in egg-laying hens [73]. Dietary supplementation with pennyroyal and savory essential oils (200, 300, and 400 ppm) did not affect food intake or body weight gain in Japanese quail [59]. Supplemented carvacrol or cinnamaldehyde (90.2 g/kg) in the diets did not alter dry matter intake or daily weight gain in lambs [40]. Similarly, when mixed with diets (2 and 4 mg/kg), cinnamaldehyde did not affect the food intake of lactating dairy cows [62]. Bees fed with essential oils from *Eupatorium buniifolium* or *Lippia graveolens* (carvacrol, thymol, and sesquiterpenes) showed no change in food intake compared with the control [68,70]. Exposure to thymol and linalool chemotypes of the essential oil of *Thymus vulgaris* did not alter food consumption in *Colossoma macropomum* juveniles [75] (Table 7 and Table 8).

#### 3.4.5. Essential Oils and Fragrant Compounds with Varied Effects

Of the 86 samples, five showed varied effects according to differences in routes of administration, dosage, and duration. Olfactory stimulation with lavender essential oil for 33 days remarkably upregulated food intake and body weight in rats. However, this effect was not observed over a similar or even longer duration when lavender essential oil was administered orally [37,64,73]. Similarly, the appetite-enhancing effects of benzylacetone, eugenol, and vanillin can be observed only after inhalation but not after intraperitoneal injection [54,61]. Trans-anethole, a main component of curry and fennel essential oils, increased food intake in mice after 1 h of inhalation at a dose of 4.5 × 10^−4^ mg/cage, while a higher dose and longer oral treatment (250 mg/kg, 10 weeks) exhibited the opposite effect [53,63,67]. (Table 9 and Table 10).

### 3.5. Mechanisms of Action

Among the 41 included publications, only 10 suggested possible underlying mechanisms of action of essential oils or compounds in appetite regulation. According to experimental data, essential oils or fragrant compounds regulate appetite-related neuropeptides, leptin resistance, autonomic nerve activity, and memory and cognitive processes to increase or reduce appetite.

#### 3.5.1. Essential Oils and Fragrant Compounds Regulate Appetite-Related Neuropeptides and Leptin Resistance

In 2013, Yamamoto et al. published the first study that examined the effects of scent stimuli on the mRNA expression of orexigenic and anorexigenic neuropeptides. Rats consuming mashed food flavored with Osmanthus essential oil showed reduced food intake after 13 days. Contemporaneously, decreases in AgRP and NPY mRNA expression and increases in *CART* and *POMC* mRNA expression were observed in these rats [34] (Table 5). Similarly, Ogawa et al. showed that trans-cinnamaldehyde, benzylacetone, and 1-phenyl-2-butanone significantly elevated food intake and NPY mRNA expression compared with the control group (Table 4). The gene expression level of POMC, an anorexigenic neuron, was attenuated simultaneously but not significantly [54]. Inhalation of patchouli essential oil or oral administration of Arq Zeera oil and its main component thymol reduced food intake and lowered elevated serum leptin levels in rats with obesity, implying that these materials reduced leptin resistance to modulate food intake [60,66] (Table 5). Yokoyama et al. demonstrated the appetite-enhancing effect of DMHF inhalation and indicated that the expression of appetite-related genes, cocaine- and amphetamine-regulated transcript prepropeptide (*Cartpt*), and angiotensinogen (*Agt*) were significantly upregulated [69] (Table 4).

#### 3.5.2. Essential Oils and Fragrant Compounds Affect Autonomic Nervous Activity

Previously, volatile compounds in grapefruit essential oil were shown to be sympathetic activators, and autonomic nerves were found to be involved in obesity genesis [76,77]. Shen et al. studied the effects of lavender and grapefruit essential oils on appetite in Wistar rats and targeted the activity of autonomic nerves as a mechanism [37,38]. Upon inhalation of lavender essential oils, increases in food intake and body weight were accompanied by reduced activity of white adipose tissue (WAT), brown adipose tissue (BAT), and adrenal sympathetic nerves, whereas the gastric vagal parasympathetic nerve showed elevated activity [37]. In contrast, grapefruit essential oil treatment exhibited opposite outcomes in rats, including decreased food consumption, excited WAT, BAT, or adrenal sympathetic nerves, and suppressed gastric parasympathetic nerve activity (PSNA) [38]. In 2013, Batubara et al. applied the same method to elucidate how *Zingiber zerumbet* essential oil and its major compound, zerumbone, control food intake in rats. Both the essential oil and zerumbone exerted an appetite-enhancing effect in rats, accompanied by a decrease in BAT SNA [46] (Table 3). Another study using citronella essential oil, R-citronella, and β-citronellol reported an increase in SNA with a reduction in appetite after inhaling β-citronellol. Interestingly, although the food intake of rats inhaling R-citronellal and citronella essential oils did not change significantly, the former reduced BAT SNA by 10% and lasted for 60 min, whereas a slight increase within 30 min was found in the latter [48] (Table 6).

#### 3.5.3. Essential Oils and Fragrant Compounds Affect Appetite via Cognitive Regulation

A 2005 study on the blowfly *Phormia regina* revealed that flies exposed to sucrose simultaneously flavored with D-limonene experienced a decreased appetite for plain sucrose that lasted for at least three days. However, this decrease was not observed in flies lacking the mushroom body, a prominent structure for primary memory in the insect brain [42,78]. To elucidate the role of memory formation in appetite regulation, which requires new protein synthesis, Nakamura et al. injected the protein synthesis inhibitor cycloheximide into the thorax of flies previously exposed to limonene and sucrose. They found that over 60% of test flies showed a proboscis extension reflex to sucrose, compared with only 20% of flies in the control group [42] (Table 6).

## 4. Discussion

To the best of our knowledge, this is the first review that presents the effects of essential oils and fragrant compounds on appetite and their underlying mechanisms. Because various diseases such as obesity, type 2 diabetes, anorexia nervosa, and bulimia nervosa are associated with dysregulated appetite and eating disorders, essential oils or fragrant compounds with appetite-regulating effects might serve as complementary and alternative medicines for treating these diseases. For example, essential oils from cinnamon, clove, or curry and their major compounds (trans-cinnamaldehyde, eugenol, and trans-anethole), which increase appetite or food intake, could be employed to treat anorexia nervosa or loss of appetite in older adults [53,54,67]. In contrast, essential oils or fragrant compounds with appetite-reducing effects, such as grapefruit and Arq Zeera essential oils, as well as related compounds such as limonene and thymol, can be used to treat bulimia nervosa, binge eating disorders, and increased appetite in patients with obesity and diabetes [38,60].

### 4.1. Routes of Administration, Doses, and Treatment Duration in Appetite Regulation

The effects of essential oils or fragrant compounds on appetite can be influenced by several factors, such as the administration route, dose, and duration of treatment. In the 41 studies included in this review, inhalation, oral administration, or mixing with diet/drink are the most frequent administration routes. These methods are common, convenient, and noninvasive. Orally administering essential oils may affect the taste of the feed, digestive processes, or immune responses [79]. Essential oils can be used as flavoring agents. A previous study showed that adding spearmint essential oils to food considerably enhances the food intake of broiler chicks [80].

Different administration methods may lead to different effects of the same essential oils or fragrant compounds on food consumption. In particular, it is likely that inhalation of essential oils or fragrant compounds is more effective at controlling appetite than other modes of delivery. Benzylacetone, eugenol, and vanillin only increased food intake by inhalation but not intraperitoneal injection [54,61]. Similarly, inhaling lavender essential oil increased appetite, but not when administered orally [37,64,73]. Orally administered drugs must pass through the gastrointestinal tract, which might affect their bioavailability and lower their effects [81]. In animal studies, the doses or concentrations of test drugs were higher, and the duration was remarkably longer when essential oils or fragrant compounds were added to the diets than when inhaled. For example, when inhaled and mixed into the diet, cinnamon essential oil showed appetite-enhancing effects. However, for dietary supplementation, the dose was 7% (*w*/*w*) of the diet for 15 days, while for inhalation, the dose was from 4.5 × 10^−4^ to 4.5 × 10^−3^ mg/cage for only 1 h of exposure [56,67]. Moreover, inhaling cinnamaldehyde for 1 h stimulated appetite, which could not be observed in cinnamaldehyde-enriched diet consumption even after 19 days of treatment [40,53,54,62,67]. This might be because inhalation provides an effective drug delivery route by delivering active pharmaceutical components to the disease target site. Inhaled essential oils or fragrant compounds can directly activate nasal olfactory chemoreceptors and olfactory signaling, which might further trigger the production of neurotransmitters and affect the neuroendocrine system and the sympathetic and parasympathetic nervous systems, resulting in psychological and appetite changes [30]. Additionally, because of the large surface area of the respiratory endothelium, drugs are rapidly absorbed compared with those accomplished via other routes of administration [82]. Low intracellular and extracellular drug-metabolizing enzyme activity can significantly improve the bioavailability of essential oils or fragrant compounds [83].

Different exposure times could also result in different appetite-related effects for certain essential oils or fragrant compounds. Inhaling linalool for 15 min daily over 33 days significantly enhanced appetite in rats, which was not replicated in mice that inhaled linalool for 1 h [37,54]. In mice that inhaled benzylacetone (7.4 × 10^−6^ mg/L) for 5, 15, 30, and 60 min, food intake increased with exposure times. On the contrary, myristicin and methyl eugenol elevated food intake in mice on the first day; however, the effects almost disappeared after the seventh day, which can be explained by the adaption process [65]. Olfactory neuron adaptation after previous exposure can decrease the sensitivity of the olfactory system to fragrant compounds. When a fragrant molecule binds to the ciliary membrane receptor, the G protein is activated, increasing the generation of cyclic adenosine monophosphate (cAMP), indirectly enhancing the intracellular concentration of calcium ions (Ca^2+^), thus transmitting information to other areas of the brain. Two secondary messengers, cAMP and Ca^2+^, which are involved in olfactory transduction processes, were found to play an important role in adaptation; however, the precise mechanism remains unclear [84]. In other cases, prolonged treatment even resulted in low toxicity with reduced weight gain and food intake when orally administered with trans-anethole for 10 weeks, while 1 h of stimulation showed an appetite-increasing effect [53,63,67].

The relative ratios of active compounds and interactions between the ingredients in essential oils can cause varied appetite-related effects. Under the same experimental conditions, cinnamon, clove, and fennel essential oils enhanced appetite at doses of 7.4 × 10^−9^ mg/cm^3^, while the effect of nutmeg essential oil only appeared at a 100-fold higher dose. This might be because cinnamon, clove, and fennel essential oils contain over 80% of the compounds that enhance appetite, while nutmeg essential oil contains only 4.25%, and the inactive compounds in nutmeg essential oil act as solvents, thereby diluting and hindering the active compounds [53,65]. In some cases, owing to the synergistic effect of constituents, appetite-regulating effects can be observed more clearly. The effect of Arq Zeera essential oil or a mixture of its main compounds (thymol and cuminaldehyde) on obese rats was better than that of thymol or cuminaldehyde alone [60].

### 4.2. Chemical Properties in Appetite Regulation

The effects of essential oils and fragrant compounds may depend on their properties, such as chemical composition and molecular structure [85,86]. Understanding molecular structure-activity connections can generate plausible analog structures with optimal solubility, efficiency, or stability in synthetic chemistry approaches. In addition, because they have similar compositions, information about the chemical skeleton of essential oils from species of the same genus can be useful for improving extraction conditions, annotating unknown analogs, and studying new skeleton structures [87,88,89].

**Figure 2:**
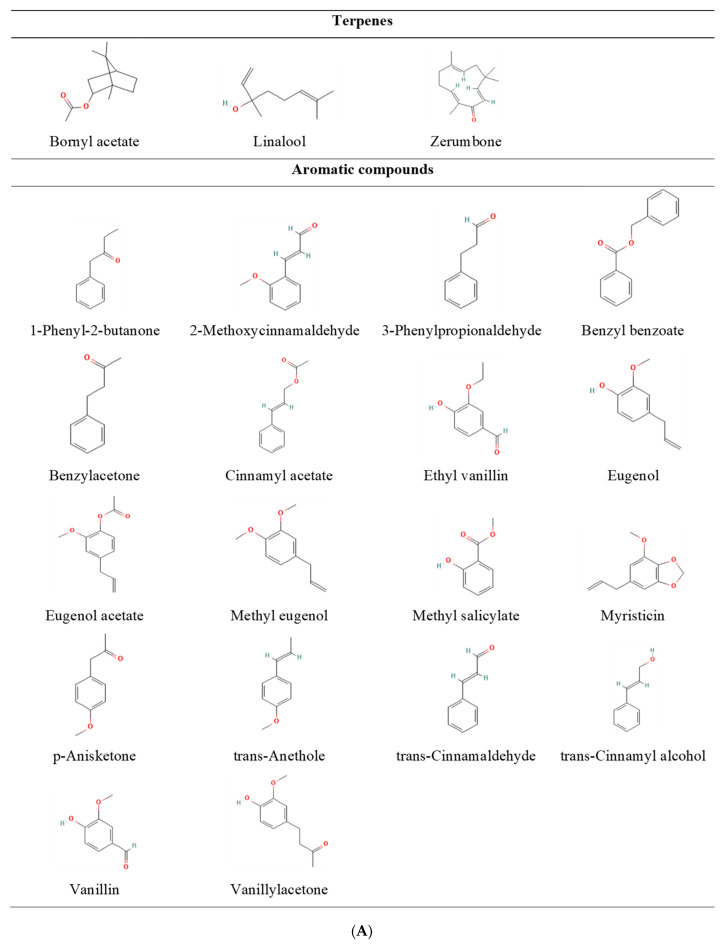

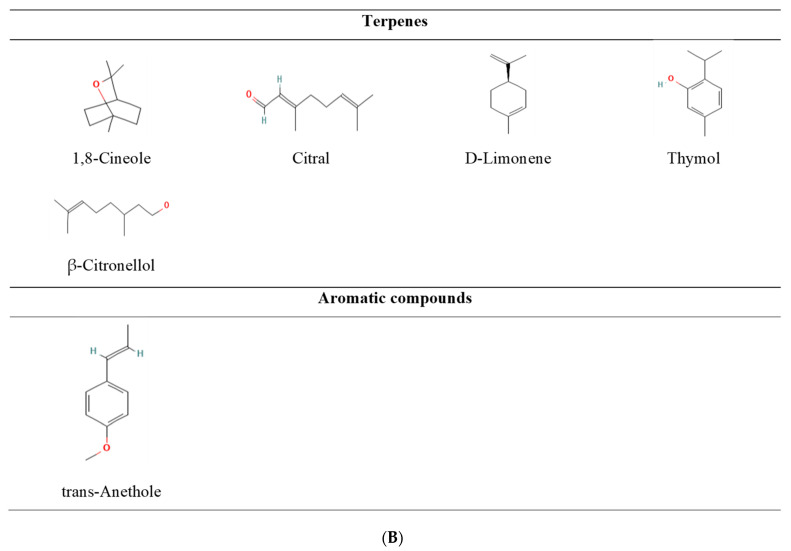

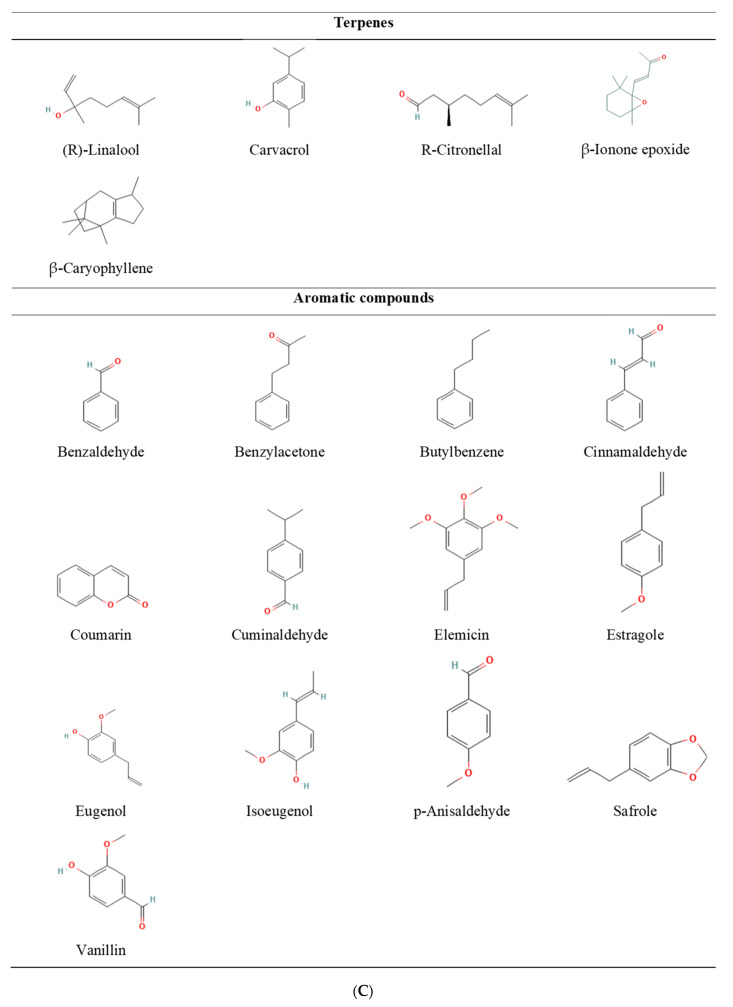

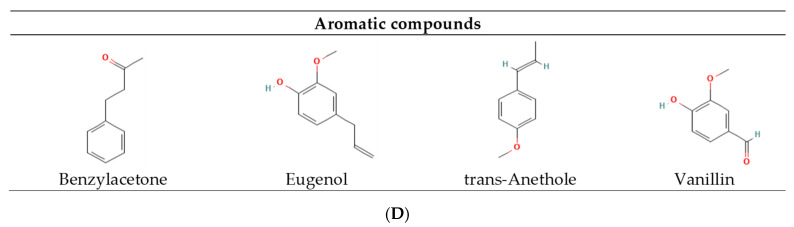
(**A**) Chemical structure of terpenes and aromatic compounds with appetite-enhancing effects. (**B**) Chemical structure of terpenes and aromatic compounds with appetite-reducing effects. (**C**) Chemical structure of terpenes and aromatic compounds having no appetite-related effects. (**D**) Chemical structure of aromatic compounds that showed varied ap-petite-related effects. Note: All chemical structures are referred from the PubChem database [100].

Essential oils commonly consist of 20–60 identified compounds, and some main components can account for up to 70% of essential oils [25]. Terpenes and aromatic compounds were the major fragrant compounds studied (Figure 2A–D). Terpene derivatives have been indicated to constitute up to 90% of essential oils and are responsible for their pharmacological and biological activities [90]. Terpenes are divided into subclasses according to their carbon units. The major terpenes are sesquiterpenes (consisting of three isoprenes with 15 carbons) and monoterpenes (consisting of two isoprenes with 10 carbons) [91]. Importantly, while some monoterpene derivatives, such as limonene, pinene, camphor, alpha-terpineol, carvone, and eucalyptol, possess cyclic properties, others do not (linalool, geraniol, citronellol, and nearly). Owing to their simple skeleton and easy synthesis, all monoterpenes are of practical importance in the biofuel, food, cosmetics, and pharmaceutical industries [92,93].

Similar to sesquiterpenes and their derivatives, these substances can be further classified as acyclic or cyclic. However, most compounds and essential oils studied for their appetite-related effects are cyclic (zerumbone, germacrene, guaiene, humulene, and caryophyllene). Owing to an electrophilic attack on double bonds leading to cyclization, the number of double bonds in cyclic sesquiterpenes is less than that in acyclic sesquiterpenes [94]. In addition, double bonds possess greater reactivity and are less stable than single bonds [95]. Thus, compared with acyclic sesquiterpenes, cyclic sesquiterpenes may exhibit superior stability, which is a priority issue for medicinal chemists. Additionally, folding of the farnesyl chain during cyclic sesquiterpene biosynthesis makes them more compact than non-cyclic ones, increasing their accessibility to receptor-binding pockets as well as their BBB permeability [94,96]. Therefore, these advantages can be the reason why researchers frequently focus on examining their effects in these studies.

Among the examined compounds, aromatic compounds with phenylpropanoid structures (such as trans-cinnamaldehyde, eugenol, eugenol acetate, and trans-anethole) significantly enhanced appetite in mice. Essential oils containing these compounds also exerted similar effects [54,67]. Fragrant compounds bearing hydroxyl or carbonyl groups on their aliphatic chains, such as benzylacetone, trans-cinnamaldehyde, and vanillylacetone, showed appetite-enhancing effects over a wide range of doses. However, these effects were not observed in compounds that either had no hydroxyl or carbonyl groups or had them but were located next to the phenyl group (butylbenzene, 6-methyl-5-hepten-2-one, benzaldehyde, estragole, and p-anisaldehyde) [54,67]. This phenomenon can be attributed to the conjugated π-bond system (the alternate between single and double bonds), an important feature of aromatic compounds [97]. The presence and distribution of double bonds and electron-withdrawing or electron-donating substituents (carbonyl, methoxyl, hydroxyl, etc.) alter the conjugation, leading to an increase or decrease in the electron density distribution of the entity molecules, affecting their stability and polarity, and thus affecting their BBB permeability and affinity towards receptors [98,99].

In another study, mice that inhaled vanillin, ethyl vanillin, or eugenol consumed significantly more food. These findings imply that the vanillyl group or its analogs, such as the 3-ethoxy-4-hydroxyphenyl group in ethyl vanillin, are present in aroma molecules that stimulate the appetite. However, even though isoeugenol contains a vanillyl group, it does not have the same appetite-stimulating properties as eugenol because its structural isomer has distinct double-bond positions. Similarly, trans-anethole and its isomer, estragole, have also been documented to exert different appetite-related effects according to the double bond distribution. Therefore, the vanillyl group and location of the double bond are predicted to affect the appetite-stimulating properties of the fragrant compounds examined [61].

### 4.3. Mechanisms of Action in Appetite Regulation

**Figure 3:**
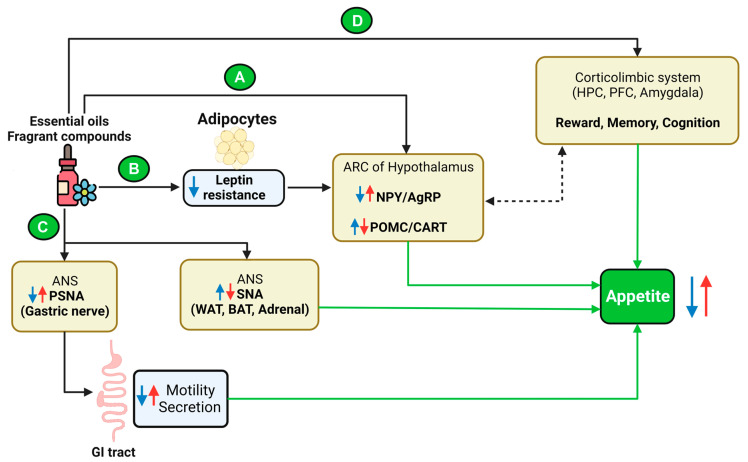
Model summarizing how essential oils or fragrant compounds (fragrant components) regulate appetite. Fragrant components regulate appetite via four pathways. (**A**) Fragrant components directly modulate the activity of NPY/AgRP and POMC/CART neurons in the ARC of the hypothalamus. Decreased NPY/AgRP mRNA expression or increased POMC/CART mRNA expression reduces appetite. (**B**) Under obesity conditions, leptin resistance develops, and food intake does not decrease despite increased leptin levels. Some essential oils can improve leptin resistance by increasing intracellular signaling pathways and transporting leptin across the BBB to the ARC, eventually decreasing food intake. (**C**) Fragrant components can excite WAT sympathetic nerves (to stimulate lipolysis), enhance BAT thermogenesis (to increase energy expenditure), and trigger adrenal SNA to induce adrenaline production, eventually decreasing food intake. Fragrant components may also, through the parasympathetic gastric nerve, inhibit gastric motility and gastric secretion to suppress appetite. (**D**) Essential oils can also regulate food intake through a scent-taste association, forming a memory or perception of a current meal, resulting in an altered amount of food intake at a later meal. Fragrant components, through the olfactory or respiratory systems, can target specific brain regions such as the HPC, hypothalamus, or amygdala to regulate appetite. Signals from the hypothalamus can also be projected onto the corticolimbic system and vice versa. Blue and red arrows represent appetite-downregulating and appetite-upregulating pathways, respectively. Black arrows represent suggested mechanisms from selected papers, and dashed black arrows represent information from other references. AgRP: agouti-related protein; ANS: autonomic nervous system; ARC: arcuate nucleus; BAT: brown adipose tissues; CART: cocaine- and amphetamine-regulated transcript; HPC: hippocampus; NPY: neuropeptide Y; PFC: the prefrontal cortex; POMC: proopiomelanocortin; PSNA: parasympathetic nerve activity; SNA: sympathetic nerve activity; WAT: white adipose tissue.

Based on the results obtained from animal studies, essential oils, and fragrant compounds may affect appetite or food consumption by regulating NPY/AgRP and CART/POMC expression, leptin resistance, SNA/PSNA activity, and cognitive processes (Figure 3).

The hypothalamus, particularly the ARC, is important in regulating the appetite-stimulating (orexigenic) and appetite-suppressing (anorexigenic) pathways to modulate food consumption. ARC neurons express orexigenic peptides (NPY and AgRP) and anorexigenic peptides (POMC and CART). The studies cited in this review established that intervention with essential oils and fragrant compounds changed the expression of NPY/AgRP and POMC/CART neurons. In studies by Yamamoto et al. and Ogawa et al., the effects of osmanthus essential oil and trans-cinnamaldehyde, benzylacetone, and 1-phenyl-2-butanone, respectively, were consistent with the general concept of these two neuron populations in controlling appetite, whereby either excited NPY/AgRP neurons or depressed CART/POMC neurons result in increased appetite, and vice versa [34,54]. However, contradictory results have been reported for DMHF administration, in which upregulated CART gene expression increased food intake [69]. No convincing explanation has been offered for this paradox; however, the localization of the target gene should be clarified after treatment. Indeed, overexpression of the CART gene in the paraventricular nucleus of the hypothalamus enhanced food intake [101]. CART neurons are expressed not only in the hypothalamus but also in the amygdala, or HPC [102]. In the DMHF study of this review, whole-brain samples of rats were used for gene expression analysis, in contrast to the hypothalamus samples used in the other two studies (Figure 3A). Frost et al. suggested that inhibition of AMP-activated protein kinase (AMPK) activity and subsequently increased acetyl-CoA carboxylase (ACC) activity elevates hypothalamic malonyl-CoA concentrations, which suppresses the expression of NPY/AgRP and stimulates POMC expression, leading to reduced food consumption [103,104]. Melissa essential oil, whose main compounds include limonene, β-citronellal, β-citral, and α-citral, activates the AMPK pathway and downregulates ACC activity to exert an antidiabetic effect [105]. Notably, the effects of limonene, β-citronellal, and citral in the selected studies also reduced food intake. Terpenoids such as citronellol, geraniol, and linalool are possible ingredients that contribute to the biological activities of pink lotus extract, which increase the phosphorylation of nuclear factor-kappa B (NF-κB), phosphatidylinositol 3-kinase (PI3K), and Akt [106]. Moreover, NPY and POMC neurotransmission could be regulated via PI3K–Akt–NF-κB signaling [107,108]. Therefore, we hypothesized that in the selected papers of this review, essential oils that have similar active components might, at least in part, modulate the activity of NPY/AgRP or CART/POMC neurons via AMPK/ACC or PI3K–Akt–NF-κB signaling pathways to regulate appetite.

ARC neurons are also first-order neurons, where peripheral metabolic signals such as leptin, insulin, ghrelin, GLP-1, and CCK are primarily sensed through their specific receptors [7,109]. Leptin is a key hormone that causes a reduction in food intake and acts as a long-term mediator to control energy balance. Normally, leptin released from adipocytes stimulates the activity of POMC neurons and suppresses the activity of AgRP/NPY neurons, leading to reduced food intake [110]. Interestingly, in the studies by Haque et al. and Hong et al., a leptin-induced decrease in food intake was not observed in obese rats [60,66]. Leptin resistance is a phenomenon whereby the brain fails to respond to leptin as it normally does, and food intake is not reduced despite higher levels of leptin [111]. The results showing that patchouli essential oil and Arq Zeera abolished the increased food consumption and serum leptin levels indicate the ability of these essential oils to mitigate leptin resistance. Both POMC and NPY neurons of the hypothalamic ARC express leptin receptors [112,113]. Because of the high permeability of the BBB around this region, leptin can easily access the ARC and bind to its receptors to activate the JAK/STAT and PI3K/AKT pathways, which are involved in appetite-down regulation [114,115,116]. However, under obesity conditions, leptin resistance develops due to impairments in intracellular signaling pathways associated with leptin receptors and leptin transport across the BBB [117]. Hence, fewer anorexigenic signals were produced, increasing appetite (Figure 3B). Although the mechanism by which essential oils such as patchouli essential oil and Arq Zeera essential oil improve leptin resistance has not been elucidated, the possibility that they act on the hypothalamus to remedy leptin resistance and enhance leptin sensitivity should not be ruled out.

Essential oils also regulate appetite via the autonomic nervous system (ANS). The ANS controls involuntary functions such as heart rate, respiratory rate, and metabolism, directly affecting energy consumption and dietary intake [118]. The sympathetic nervous system can constrict blood vessels in the digestive tract, decreasing digestion and appetite, whereas the parasympathetic nervous system relaxes digestive tract muscles to enhance digestion and appetite [119,120]. Although these two systems are often antagonistic to each other, they work together to regulate body functions and maintain homeostasis [121]. Shen et al. and Batubara et al. reported that either suppressed BAT, WAT, and adrenal sympathetic nerves or enhanced gastric parasympathetic nerves were observed with an increased appetite, while the opposite trends were observed in appetite downregulation [37,38,46,48] (Figure 3C). Shen et al. explained that grapefruit essential oil might stimulate lipolysis by stimulating the WAT sympathetic nerves, enhancing BAT heat production via activating uncoupling protein 1, and stimulating adrenaline production by exciting adrenal SNA, which eventually decreases food intake. In contrast, the lavender essential oil was supposed to exert the opposite effect and enhance appetite. The sympathetic nerves innervating BATs and WATs could modulate appetite by regulating the secretion of noradrenaline (NA), a neurotransmitter that normally acts in hypothalamic sites to regulate feeding [122,123]. Moreover, NA is also one of the key physiological mediators of BAT heat production, which directly affects the energy expenditure of the body and food intake [124,125,126]. Regarding the gastric parasympathetic nerves, grapefruit might have downregulated appetite via parasympathetic control in the vagus nerve, inhibiting gastric motility and gastric juice secretion.

Decision-making for a certain food depends on one’s experience with that food. This phenomenon has been related to food reward and cognitive processes, or “memory for recent eating” [127,128]. Many studies have highlighted the critical role of episodic memory in the onset and termination of normal meals and suggested that recalling the memory of a recent meal can result in an altered amount of food intake at a later meal [9,128,129,130]. Sensory signals, such as sight, smell, and taste, are proposed to override satiety signals in food intake maintenance. This is accomplished by integrating sensory signals in the nucleus tractus solitarius of the brainstem and relaying them to corticolimbic reward centers in the brain, such as the HPC, amygdala, and nucleus accumbens, which perform appetite-regulating functions [131,132,133]. The HPC has been reported to encode, store, and recall memories of individual experiences, and leptin receptors also exist at high levels throughout this brain region [134,135]. Moreover, hippocampal neurons send extensive projections to the hypothalamus for energy homeostasis and receive neural signals for calorie consumption [136,137] (Figure 3D). Several essential oils that were found to act on the hypothalamus, HPC, and amygdala include trans-cinnamaldehyde, benzylacetone, 1-phenyl-2-butanone, lavender essential oil, and *Cananga odorata* essential oil [54,138,139]. Another study showed that the inhalation of peppermint essential oil ameliorated impaired memory in mice by normalizing the excited state of neurons in the hippocampal CA1 region [140]. Nakamura et al. demonstrated the involvement of cognition and memory in appetite regulation. Flies that previously experienced food with an unpleasant odor (D-limonene) maintained a significantly reduced appetite for that food for at least three days. This could be explained by the unpleasant odor-induced memory of the food that encouraged avoidance [42]. Indeed, flies that lacked the mushroom body, which is required for memory formation, did not show any reduction in sucrose appetite, while cycloheximide, a protein synthesis inhibitor, improved appetite loss in limonene-treated flies [42,141,142]. These data indicate that cycloheximide impedes the appetite-reducing effect of limonene by inhibiting protein synthesis, thereby indirectly suggesting that appetite modulation is mediated via the formation of memory.

### 4.4. Quality of Selected Studies

Most of the selected studies meet the basic requirements of a research paper: sufficiently providing information about the research subject (sex, species, age, weight), clearly describing the experimental method, essential oil preparation, and treatment (dosage, duration, route of administration). However, not all studies provide the scientific names of the test essential oils as well as an analysis of their chemical components, leading to inconsistencies in the contents of Table 2, Table 3, Table 5, Table 7 and Table 9. Essential oils in the same genus but different species may have different chemical compositions and properties; hence, a provision of the essential oil’s scientific name is of great necessity for other researchers to prepare the experiments and reproduce consistent results.

Out of 41 studies, one study scored 10 points and five studies scored 9 points, and they were all published from 2016 to 2022, while the studies that scored 6 and 7 were distributed in the 2003 to 2015 period. Overall, in terms of our 10-item checklist, the quality of the selected studies has gradually improved. However, a notable limitation is that only 10 of 41 publications mention the mechanisms underlying the stimulating or suppressing effects of the test samples, and not all have been proven experimentally. Furthermore, from 2016 to 2022, the number of mechanism-mentioned studies is 4, necessitating a focus on these issues in future research. Most selected studies used only food intake or food consumption as a key indicator of appetite. It is proposed that other biomarkers, such as dopamine, orexin, gamma-aminobutyric acid, ghrelin, GLP-1, or CCK, can also be used to assess appetite [7,143]. In our opinion, it is a critical point, but not valid in all cases. An increase or decrease in food intake should be the most convincing evidence for assessing one’s appetite, rather than just changes in hormone and neurotransmitter levels, which are normally regulated by various factors. That could be the reason why 41 selected studies did not use these markers as the main indicators for appetite. However, we agree that future studies should consider performing additional animal behavioral tests in evaluating appetites, such as the two-bottle choice test or conditioned place preference test [144,145]. Besides, in this review, the terms “food intake” and “body weight” run together at times. It is of great importance to notice that body weight changes were not used to assess appetite in the included studies. For example, in studies [47,63], body weight remained constant despite a decrease in food intake. Therefore, body weight changes do not necessarily reflect appetite.

Finally, nearly all studies in the review overlooked the possible toxicity of essential oils and compounds when applied in animal models. Therefore, despite the appetite-regulating potential pointed out in in vivo models, further research confirming the safety of these essential oils or fragrant compounds is needed before investigating clinical studies or drug development.

## 5. Conclusions

Essential oils that increase appetite include lavender, fennel, black pepper, vanilla, curry, cinnamon, clove, nutmeg, cang-ai, *Zingiber zerumbet*, and *Amomum villosum* Lour. Contrarily, grapefruit, peppermint, slique essence, thyme, arq zeera, patchouli, pine, *Carvi aetheroleum, Citrus aurantifolia, Osmanthus fragrans,* and *Croton zehntneri* essential oils suppressed appetite. Fragrant compounds, including linalool, zerumbone, eugenol, methyl eugenol, eugenol acetate, trans-cinnamaldehyde, cinnamyl acetate, trans-cinnamyl alcohol, 1-phenyl-2-butanone, benzylacetone, bornyl acetate, vanillin, vanillylacetone, ethyl vanillin, myristicin, 2-methoxycinnamaldehyde, 3-phenylpropionaldehyde, benzyl benzoate, methyl salicylate, p-anisketone, and DMHF, increased appetite. In contrast, citral, limonene, D-limonene, β-citronellol, thymol, and 1,8-cineole decreased appetite. Notably, trans-anethole exhibited both appetite-stimulating and appetite-suppressing effects. These essential oils and fragrant compounds control appetite via changes in NPY/AgRP and CART/POMC mRNA expression, modulation of leptin release, and sympathetic and parasympathetic nerve activity; however, processes related to memory and cognition remain to be confirmed. Further research is necessary for therapeutic applications because the effect on appetite differs based on the concentration, period, and route of administration.

## Figures and Tables

**Table 1:** Quality assessment of included studies.

No.	Authors	(1)	(2)	(3)	(4)	(5)	(6)	(7)	(8)	(9)	(10)	Score	References
1	Ress et al., 2003	1	1	1	0	1	1	0	0	0	1	6	[33]
2	Shen et al., 2005	1	1	1	0	1	0	1	1	0	1	7	[37]
3	Shen et al., 2005	1	0	1	0	1	0	1	1	0	1	6	[38]
4	Schöne et al., 2006	0	0	1	1	1	0	0	0	0	1	4	[39]
5	Chaves et al., 2008	1	1	1	0	1	0	1	0	0	1	6	[40]
6	Munakata et al., 2008	0	0	1	0	1	1	0	0	0	1	4	[41]
7	Nakamura et al., 2008	1	1	1	0	1	1	0	1	0	1	7	[42]
8	Suanarunsawat et al., 2009	1	1	1	1	1	1	1	0	0	1	8	[43]
9	Asnaashari et al., 2010	1	1	1	0	0	0	1	0	1	1	6	[44]
10	Khodambashi et al., 2012	1	1	1	1	1	1	0	0	0	1	7	[45]
11	Batubara et al., 2013	1	1	1	0	1	0	0	1	1	1	7	[46]
12	Caldas et al., 2013	1	1	1	0	1	0	1	0	1	1	7	[47]
13	Yamamoto et al., 2013	1	0	1	0	1	1	0	1	1	1	7	[34]
14	Batubara et al., 2015	1	1	1	0	0	0	0	1	1	1	6	[48]
15	Escobar et al., 2015	1	1	1	1	1	1	1	0	0	1	8	[49]
16	Khosravinia, 2015	1	1	1	1	1	1	0	0	0	1	7	[50]
17	Dahham et al., 2016	1	0	1	0	1	1	1	0	1	1	7	[51]
18	Firmin et al., 2016	1	0	1	0	1	1	0	0	0	1	5	[52]
19	Ogawa and Ito, 2016	1	1	1	1	1	1	1	0	1	1	9	[53]
20	Ogawa and Ito, 2016	1	1	1	1	1	1	1	1	1	1	10	[54]
21	Ogawa and Ito, 2016	1	1	1	0	1	1	1	0	1	1	8	[55]
22	Ornaghi et al., 2017	1	1	1	0	1	0	1	0	1	1	7	[56]
23	Walia et al., 2017	1	1	1	0	0	0	0	0	0	1	4	[57]
24	Zhang et al., 2017	1	1	1	1	1	1	0	0	1	1	8	[58]
25	Dehghani et al., 2018	1	1	1	0	1	1	0	0	0	1	6	[59]
26	Haque and Ansari, 2018	1	1	1	1	1	0	1	1	1	1	9	[60]
27	Ogawa et al., 2018	1	1	1	1	1	1	1	0	1	1	9	[61]
28	Chapman et al., 2019	1	1	1	0	1	1	1	0	0	1	7	[62]
29	Coelho-de-Souza et al., 2019	1	1	1	0	1	0	1	0	1	1	7	[63]
30	Mekonnen et al., 2019	1	1	1	0	1	1	1	0	1	1	8	[64]
31	Ogawa and Ito, 2019	1	1	1	1	1	1	1	0	1	1	9	[65]
32	Hong et al., 2020	1	1	1	0	1	1	0	1	1	1	8	[66]
33	Ogawa et al., 2020	1	1	1	1	1	1	1	0	1	1	9	[67]
34	Rossini et al., 2020	1	1	1	0	1	0	0	0	0	1	5	[68]
35	Yokoyama et al., 2020	1	1	1	0	1	1	0	1	1	1	8	[69]
36	Canche-Colli et al., 2021	1	1	1	1	1	0	0	0	1	1	7	[70]
37	Lu et al., 2021	0	1	1	0	1	1	1	0	1	1	7	[71]
38	Rafferty and Lamont, 2021	1	1	1	0	1	0	0	0	1	1	6	[72]
39	Torki et al., 2021	1	1	1	0	1	1	0	0	1	1	7	[73]
40	Zhang et al., 2021	0	0	1	1	1	0	1	0	1	1	6	[74]
41	Boaventura et al., 2022	1	1	1	0	1	0	1	0	1	1	7	[75]

Criteria of quality assessment: (1) description of the sampling procedure or compound manufacturer; (2) mention of essential oil compositions; (3) detailed description of interventions; (4) use of positive controls; (5) specific mention of strains/species of animals used; (6) mention of test subject age; (7) mention of test subject weight; (8) mention or explanation of underlying mechanisms; (9) statement of potential conflict of interests; and (10) peer-reviewed publication.

**Table 2:** Effects of essential oils on appetite in clinical studies.

No.	Essential Oil	Major Compounds	Route of Administration	Dose	Duration	Effects on Appetite	Mechanism	References
1	Black pepper essential oil	n/a	Intranasal	100 µL/filter paper stick	1 min	Increase	n/a	[41]
2	Vanilla essential oil	n/a	Inhalation	Two drops (~0.1 mL)/petri dish	n/a	Increase	n/a	[52]
3	Slique Essence	n/a	Inhalation	Two drops (~0.1 mL)/petri dish	n/a	Decrease	n/a	[52]

n/a, not applicable.

**Table 3:** Essential oils with appetite-enhancing effects in animal models.

No.	Essential Oil	Major Compounds	Route of Administration	Dose	Duration	Species	Mechanism	References
1	Lavender essential oil	Linalool 1,8-Cineole (Eucalyptol) Camphor	Inhalation	100,000× dilution in water	15 min/day, 33 days	Wistar rats	Decrease WAT, BAT, and adrenal SNA; increase gastric PSNA	[37]
2	Fennel essential oil	trans-Anethole Fenchone	Mix with diet	100 mg/kg of diet	3 weeks	Pietrain × (Landrace × Large White) pigs	n/a	[39]
3	*Zingiber zerumbet* essential oil	Zerumbone	Inhalation	100× dilution in water	5 weeks	Sprague Dawley rats	Decrease BAT SNA	[46]
4	Curry essential oil	trans-Anethole 2-Methyl-3-phenylpropanal	Inhalation	4.5 × 10^−4^ mg/cage	1 h	ddY mice	n/a	[53]
5	Cinnamon essential oil	trans-Cinnamaldehyde trans-2-Methoxycinnamaldehyde	Mix with diet	7% (*w*/*w*) of diet	15 days	Crossbred bulls	n/a	[56]
6	Clove essential oil	Eugenol Eugenol acetate	Mix with diet	3.5% (*w*/*w*) of diet	15 days	Crossbred bulls	n/a	[56]
7	*Amomum villosum* Lour. essential oil	Bornyl acetate Camphor	Oral	8, 16, 32 mg/kg	12 days	Sprague Dawley rats	n/a	[58]
8	Nutmeg essential oil	Sabinene α-Pinene	Inhalation	7.4 × 10^−7^ mg/cm^3^	1 h	ddY mice	n/a	[65]
9	Cinnamon essential oil	trans-Cinnamaldehyde trans-2-Methoxycinnamaldehyde	Inhalation	4.5 × 10^−4^~4.5 × 10^−3^ mg/cage	1 h	ddY mice	n/a	[67]
10	Clove essential oil	Eugenol Eugenol acetate	Inhalation	4.5 × 10^−4^~4.5 × 10^−3^ mg/cage	1 h	ddY mice	n/a	[67]
11	Fennel essential oil	trans-Anethole Fenchone	Inhalation	4.5 × 10^−4^~4.5 × 10^−3^ mg/cage	1 h	ddY mice	n/a	[67]
12	Cang-ai essential oil	Eugenol 1,8-Cineole	Oral	4.6 μg/kg/day	28 days	Sprague Dawley rats	n/a	[74]

BAT, brown adipose tissue; n/a, not applicable; PSNA, parasympathetic nerve activity; SNA, sympathetic nerve activity; WAT, white adipose tissue.

**Table 4:** Fragrant compounds with appetite-enhancing effects in animal models.

No.	Fragrant Compounds	Route of Administration	Dose	Duration	Model	Mechanism	References
1	Linalool	Inhalation	5000× dilution in water	33 days	Wistar rats	Decrease adrenal SNA, increase gastric PSNA	[37]
2	Zerumbone	Inhalation	100× dilution in water	5 weeks	Sprague Dawley rats	Decrease BAT SNA	[46]
3	Eugenol	Inhalation	4.5 × 10^−4^ mg/cage	1 h	ddY mice	n/a	[53]
4	Mixture of trans-cinnamaldehyde, eugenol, trans-anethole (1:2.6:5.6)	Inhalation	4.5 × 10^−5^ mg/cage	1 h	ddY mice	n/a	[53]
5	trans-Anethole	Inhalation	4.5 × 10^−4^ mg/cage	1 h	ddY mice	n/a	[53]
6	trans-Cinnamaldehyde	Inhalation	4.5 × 10^−4^ mg/cage	1 h	ddY mice	n/a	[53]
7	1-Phenyl-2-butanone	Inhalation	4.5 × 10^−4^ mg/cage	1 h	ddY mice	Increase NPY mRNA expression	[54]
8	Benzylacetone	Inhalation	4.5 × 10^−4^ mg/cage	1 h	ddY mice	Increase NPY mRNA expression	[54]
9	trans-Cinnamaldehyde	Inhalation	4.5 × 10^−4^ mg/cage	1 h	ddY mice	Increase NPY mRNA expression	[54]
10	Bornyl acetate	Oral	2, 4, 8 mg/kg	12 days	Sprague Dawley rats	n/a	[58]
11	Ethyl vanillin	Inhalation	4.5 × 10^−4^~4.5 × 10^−3^ mg/cage	1 h	ddY mice	n/a	[61]
12	Eugenol	Inhalation	4.5 × 10^−4^~2.5 × 10^−3^ mg/cage	1 h	ddY mice	n/a	[61]
13	Vanillin	Inhalation	4.5 × 10^−5^~4.5 × 10^−3^ mg/cage	1 h	ddY mice	n/a	[61]
14	Benzylacetone	Inhalation	7.4 × 10^−8^~7.4 × 10^−2^ mg/cm^3^	5~60 min	ddY mice	n/a	[65]
15	Methyl eugenol	Inhalation	7.4 × 10^−9^ mg/cm^3^	1 h	ddY mice	n/a	[65]
16	Myristicin	Inhalation	7.4 × 10^−9^ mg/cm^3^	1 h	ddY mice	n/a	[65]
17	Vanillylacetone	Inhalation	7.4 × 10^−10^~7.4 × 10^−7^ mg/cm^3^	1 h	ddY mice	n/a	[65]
18	2-Methoxycinnamaldehyde	Inhalation	4.5 × 10^−5^~4.5 × 10^−4^ mg/cage	1 h	ddY mice	n/a	[67]
19	3-Phenylpropionaldehyde	Inhalation	4.5 × 10^−4^~4.5 × 10^−3^ mg/cage	1 h	ddY mice	n/a	[67]
20	Benzyl benzoate	Inhalation	4.5 × 10^−6^~2.5 × 10^−3^ mg/cage	1 h	ddY mice	n/a	[67]
21	Benzylacetone	Inhalation	4.5 × 10^−4^ mg/cage	1 h	ddY mice	n/a	[67]
22	Cinnamyl acetate	Inhalation	4.5 × 10^−5^~4.5 × 10^−4^ mg/cage	1 h	ddY mice	n/a	[67]
23	Eugenol	Inhalation	4.5 × 10^−4^ mg/cage	1 h	ddY mice	n/a	[67]
24	Eugenol acetate	Inhalation	1.1 × 10^−3^~4.5 × 10^−3^ mg/cage	1 h	ddY mice	n/a	[67]
25	Methyl salicylate	Inhalation	4.5 × 10^−4^~2.5 × 10^−3^ mg/cage	1 h	ddY mice	n/a	[67]
26	Mixture of eugenol and eugenol acetate (2:1)	Inhalation	4.5 × 10^−4^ mg/cage	1 h	ddY mice	n/a	[67]
27	p-Anisketone	Inhalation	4.5 × 10^−4^~2.5 × 10^−3^ mg/cage	1 h	ddY mice	n/a	[67]
28	trans-Anethole	Inhalation	4.5 × 10^−4^ mg/cage	1 h	ddY mice	n/a	[67]
29	trans-Cinnamaldehyde	Inhalation	4.5 × 10^−4^~4.5 × 10^−3^ mg/cage	1 h	ddY mice	n/a	[67]
30	trans-Cinnamyl alcohol	Inhalation	4.5 × 10^−4^~4.5 × 10^−2^ mg/cage	1 h	ddY mice	n/a	[67]
31	2,5-Dimethyl-4-hydroxy-3(2H)-furanone (DMHF)	Inhalation	5.7 mg/L water	6 weeks	Wistar rats	Increase mRNA expression of *Cartpt*, and *Agt*	[69]

*Agt*, angiotensinogen; BAT, brown adipose tissue; *Cartpt*, cocaine- and amphetamine-regulated transcript prepropeptide; n/a, not applicable; NPY, neuropeptide Y; PSNA, parasympathetic nerve activity; SNA, sympathetic nerve activity.

**Table 5:** Essential oils with appetite-reducing effects in animal models.

No.	Essential Oil	Major Compounds	Route of Administration	Dose	Duration	Species	Mechanism	References
1	Grapefruit essential oil	Limonene	Inhalation		15 min, 3 times/week for 6 weeks	Wistar rats, C57BL/6J mice	Increase WAT, BAT, and adrenal SNA; decrease gastric PSNA	[38]
2	Caraway (*Carvi aetheroleum*) essential oil	Limonene Carvone	Mix with diet	100 mg/kg of diet	3 weeks	Pietrain × (Landrace × Large White) pigs	n/a	[39]
3	*Citrus aurantifolia* essential oil	D-Limonene α-Terpineol	Subcutaneous injection	125, 250, 500 mg/kg	45 days	Mice	n/a	[44]
4	Peppermint essential oil	(L)-menthol (L)-menthone	Mix with diet	400, 500 mg/kg	6 weeks	Ross 308 Broilers	n/a	[45]
5	*Osmanthus fragrans* essential oil	n/a	Inhalation	100 μL/filter paper	23 days	Wistar rats	Decrease mRNA expression of AgRP and NPY, increase mRNA expression of CART and POMC	[34]
6	FormaXOL	n/a	Mix with diet	4 kg/ton diet	28 days	Pigs	n/a	[57]
7	Thyme essential oil	Thymol Durenol	Mix with diet	400 ppm	35 days	Japanese quail	n/a	[59]
8	Arq zeera (*Trachyspermum ammi* L., *Zingiber officinale* Roxb., *Carum carvi* L., and *Cuminum cyminum* L)		Oral	7.75 mg/kg	Twice per day for 4 weeks	Wistar rats	Lower the elevated serum leptin level (reduced leptin resistance) in obese rats	[60]
9	*Croton zehntneri* essential oil	trans-Anethole Estragole	Oral	250 mg/kg	10 weeks	Wistar rats	n/a	[63]
10	Patchouli essential oil	α-Patchoulene β-Patchoulene	Inhalation	0.3% or 1%	30 min for 6 or 12 weeks	Sprague Dawley rats	Lower the elevated serum leptin level (reduced leptin resistance) in obese rats	[66]
11	Pine essential oil	n/a	Mix with diet	2% (*w*/*w*) of diet	n/a	Western grey kangaroos	n/a	[72]

AgRP, agouti-related protein; BAT, brown adipose tissue; CART, cocaine- and amphetamine-regulated transcript; n/a, not applicable; NPY, neuropeptide Y; POMC, proopiomelanocortin; PSNA, parasympathetic nerve activity; SNA, sympathetic nerve activity; WAT, white adipose tissue.

**Table 6:** Fragrant compounds with appetite-reducing effects in animal models.

No.	Fragrant Compounds	Route of Administration	Dose	Duration	Model	Mechanism	References
1	Citral	Mix with diet	3900, 7800, 15,600, 31,300 ppm	14 weeks	F344/N rats	n/a	[33]
2	Limonene	Inhalation	5000× dilution in water	6 weeks	Wistar rats, C57BL/6J mice	Increase adrenal SNA, decrease gastric PSNA	[38]
3	D-Limonene	Inhalation	Flow rate: 200 mL/min	10 min	Blowflies	n/a	[42]
4	β-Citronellol	Inhalation	100× dilution in water	35 days	Sprague Dawley rats	Increase BAT SNA	[48]
5	Thymol	Oral	12 mg/kg	Twice per day for 4 weeks	Wistar rats	Lower the elevated serum leptin level (reduced leptin resistance) in obese rats	[60]
6	trans-Anethole	Oral	250 mg/kg	10 weeks	Wistar rats	n/a	[63]
7	1,8-Cineole	Mix with diet	2% (*w*/*w*) of diet	n/a	Western grey kangaroos	n/a	[72]

BAT, brown adipose tissue; n/a, not applicable; PSNA, parasympathetic nerve activity; SNA, sympathetic nerve activity.

**Table 7:** Essential oils showing no appetite-related effects in animal models.

No.	Essential Oil	Major Compounds	Route of Administration	Dose	Duration	Species	Mechanism	References
1	*Ocimum sanctum* L. essential oil	Eugenol Methyl eugenol	Mix with diet	80 µL/kg bw/day	3 weeks	Wistar rats	n/a	[43]
2	*Hyptis martiusii* Benth. essential oil	1,8-Cineole δ-3-Carene	Oral	100, 500 mg/kg	30 days	Swiss mice	n/a	[47]
3	Citronella essential oil	R-Citronellal Neryl acetate	Inhalation	100× dilution in water		Sprague Dawley rats	n/a	[48]
4	*Minthostachys verticillata* essential oil	Pulegone Menthone	Mix with diet	1, 4, 7 g/kg feed	90 days	Wistar rats	n/a	[49]
5	*Satureja khuzistanica* essential oil	Carvacrol p-Cymene	Mix with water	200, 300, 400, and 500 mg/L in water	42 days	Arian broiler chicks	n/a	[50]
6	*Aquilaria crassna* essential oil	n/a	Oral	100, 500 mg/kg	28 days	Swiss mice	n/a	[51]
7	Ginger essential oil	Geranial Neral	Inhalation	4.5 × 10^−4^ mg/cage	1h	ddY mice	n/a	[53]
8	Geranium essential oil	n/a	Inhalation	4.5 × 10^−3^ mg/cage	1h	ddY mice	n/a	[54]
9	Pennyroyal essential oil	Pulegone 3,3′-dimenthol	Mix with diet	200, 300, and 400 ppm	35 days	Japanese quail	n/a	[59]
10	Savory essential oil	Thymol 4,4′-diapophytoene	Mix with diet	200, 300, and 400 ppm	35 days	Japanese quail	n/a	[59]
11	Lavender essential oil	Linalool 1,8-Cineole (Eucalyptol) Camphor	Oral	2000 mg/kg	21 days	Swiss albino mice	n/a	[64]
12	*Eupatorium buniifolium* essential oil	α-pinene (E)-β-guaiene	Oral	300, 3000, 6000 ppm	12 days	*Apis mellifera* bees	n/a	[68]
13	Carvacrol essential oil from *Lippia graveolens*	Carvacrol p-Cymene	Mix with diet	1% (*w*/*w*) of diet	9~12 days	*Apis mellifera* bees	n/a	[70]
14	Sesquiterpenes essential oil from *Lippia graveolens*	β-Caryophyllene α-Humulene	Mix with diet	1% (*w*/*w*) of diet	9~12 days	*Apis mellifera* bees	n/a	[70]
15	Thymol essential oil from *Lippia graveolens*	Thymol β-Caryophyllene	Mix with diet	1% (*w*/*w*) of diet	9~12 days	*Apis mellifera* bees	n/a	[70]
16	Lavender essential oil	Linalool 1,8-Cineole (Eucalyptol) Camphor	Mix with diet	250 mg/kg diet	14 weeks	Lohmann LSL-Lite laying hens	n/a	[73]
17	*Mentha spicata* essential oil	Carvone Limonene	Mix with diet	250 mg/kg diet	14 weeks	Lohmann LSL-Lite laying hens	n/a	[73]
18	Linalool essential oil from *Thymus vulgaris*	Linalool Carvacrol	Mixed in aquarium	100 mg/L	n/a	*Colossoma macropomum*	n/a	[75]
19	Thymol essential oil from *Thymus vulgaris*	Thymol p-Cymene	Mixed in aquarium	50 mg/L	n/a	*Colossoma macropomum*	n/a	[75]

bw: body weight; n/a: not applicable.

**Table 8:** Fragrant compounds showing no appetite-related effects in animal models.

No.	Fragrant Compounds	Route of Administration	Dose	Duration	Model	Mechanism	References
1	Carvacrol	Mix with diet	0.2 g/kg	7 days	Canadian Arcott lambs	n/a	[40]
2	Cinnamaldehyde	Mix with diet	0.2 g/kg	7 days	Canadian Arcott lambs	n/a	[40]
3	R-Citronellal	Inhalation	100× dilution in water	35 days	Sprague Dawley rats	n/a	[48]
4	Estragole	Inhalation	4.5 × 10^−4^ mg/cage	1 h	ddY mice	n/a	[53]
5	Safrole	Inhalation	4.5 × 10^−4^ mg/cage	1 h	ddY mice	n/a	[53]
6	β-Caryophyllene	Inhalation	4.5 × 10^−4^ mg/cage	1 h	ddY mice	n/a	[53]
7	(R)-Linalool	Inhalation	4.5 × 10^−5^ mg/cage	1 h	ddY mice	n/a	[54]
8	6-Methyl-5-hepten-2-one	Inhalation	4.5 × 10^−4^ mg/cage	1 h	ddY mice	n/a	[54]
9	Benzaldehyde	Inhalation	4.5 × 10^−5^ mg/cage	1 h	ddY mice	n/a	[54]
10	Benzylacetone	Intraperitoneal injection	0.01–1 μg/kg	1 h	ddY mice	n/a	[54]
11	Butylbenzene	Inhalation	4.5 × 10^−4^ mg/cage	1 h	ddY mice	n/a	[54]
12	Cuminaldehyde	Oral	6 mg/kg	Twice per day for 4 weeks	Wistar rats	n/a	[60]
13	Eugenol	Intraperitoneal injection	0.01–1 μg/kg	1 h	ddY mice	n/a	[61]
14	Isoeugenol	Inhalation	4.5 × 10^−5^–4.5 × 10^−3^ mg/cage	1 h	ddY mice	n/a	[61]
15	Safrole	Inhalation	4.5 × 10^−5^–4.5 × 10^−3^ mg/cage	1 h	ddY mice	n/a	[61]
16	Vanillin	Intraperitoneal injection	0.01–1 μg/kg	1 h	ddY mice	n/a	[61]
17	Cinnamaldehyde	Mix with diet	2, 4 mg/kg of bw	19 days	Holsteinian dairy cows	n/a	[62]
18	Elemicin	Inhalation	7.4 × 10^−10^–7.4 × 10^−8^ mg/cm^3^	1 h	ddY mice	n/a	[65]
19	Estragole	Inhalation	4.5 × 10^−5^–4.5 × 10^−3^ mg/cage	1 h	ddY mice	n/a	[67]
20	Coumarin	Inhalation	4.5 × 10^−5^–4.5 × 10^−3^ mg/cage	1 h	ddY mice	n/a	[67]
21	p-Anisaldehyde	Inhalation	4.5 × 10^−5^–4.5 × 10^−3^ mg/cage	1 h	ddY mice	n/a	[67]
22	β-Ionone epoxide	Mix with diet	20, 40, 80 mg/kg bw/day	90 days	Sprague Dawley rats	n/a	[71]

bw: body weight; n/a: not applicable.

**Table 9:** Essential oils showing varied appetite-related effects in animal models.

No.	Essential Oil	Major Compounds	Route of Administration	Dose	Duration	Species	Effect on Appetite	Mechanism	References
1	Lavender essential oil	Linalool 1,8-Cineole (Eucalyptol) Camphor	Inhalation	100,000× dilution in water	15 min/day, 33 days	Wistar rats	Increase	Decrease WAT, BAT, and adrenal SNA; increase gastric PSNA	[37]
			Oral	2000 mg/kg	21 days	Swiss albino mice	No effect	n/a	[64]
			Mix with diet	250 mg/kg diet	14 weeks	Lohmann LSL-Lite laying hens	No effect	n/a	[73]

BAT, brown adipose tissue; n/a, not applicable; PSNA, parasympathetic nerve activity; SNA, sympathetic nerve activity; WAT, white adipose tissue.

**Table 10:** Fragrant compounds showing varied appetite-related effects in animal models.

No.	Fragrant Compounds	Route of Administration	Dose	Duration	Model	Effects on Appetite	Mechanism	References
1	Benzylacetone	Inhalation	4.5 × 10^−4^ mg/cage	1 h	ddY mice	Increase	Increase NPY mRNA expression	[54]
		Intraperitoneal injection	0.01–1 μg/kg	1 h	ddY mice	No effect	n/a	[54]
		Inhalation	7.4 × 10^−8^–7.4 × 10^−2^ mg/cm^3^	5–60 min	ddY mice	Increase	n/a	[55]
		Inhalation	7.4 × 10^−8^–7.4 × 10^−2^ mg/cm^3^	5–60 min	ddY mice	Increase	n/a	[65]
		Inhalation	4.5 × 10^−4^ mg/cage	1 h	ddY mice	Increase	n/a	[67]
2	Eugenol	Inhalation	4.5 × 10^−4^ mg/cage	1 h	ddY mice	Increase	n/a	[53]
		Inhalation	4.5 × 10^−4^–2.5 × 10^−3^ mg/cage	1 h	ddY mice	Increase	n/a	[61]
		Intraperitoneal injection	0.01–1 μg/kg	1 h	ddY mice	No effect	n/a	[61]
		Inhalation	4.5 × 10^−4^ mg/cage	1 h	ddY mice	Increase	n/a	[67]
3	trans-Anethole	Inhalation	4.5 × 10^−4^ mg/cage	1 h	ddY mice	Increase	n/a	[53]
		Oral	250 mg/kg	10 weeks	Wistar rats	Decrease	n/a	[63]
		Inhalation	4.5 × 10^−4^ mg/cage	1 h	ddY mice	Increase	n/a	[67]
4	Vanillin	Inhalation	4.5 × 10^−5^–4.5 × 10^−3^ mg/cage	1 h	ddY mice	Increase	n/a	[61]
		Intraperitoneal injection	0.01–1 μg/kg	1 h	ddY mice	No effect	n/a	[61]

n/a, not applicable; NPY, neuropeptide Y.

## Data Availability

Data sharing is not applicable to this article as no new data were created or analyzed in this study.
